# Targeting of lysosomal-bound protein mEAK-7 for cancer therapy

**DOI:** 10.3389/fonc.2024.1375498

**Published:** 2024-03-12

**Authors:** Insoon Chang, Yi-Ling Loo, Jay Patel, Joe Truong Nguyen, Jin Koo Kim, Paul H. Krebsbach

**Affiliations:** ^1^ Section of Endodontics, Division of Regenerative and Reconstructive Sciences, School of Dentistry, University of California, Los Angeles, Los Angeles, CA, United States; ^2^ School of Dentistry, University of California, Los Angeles, Los Angeles, CA, United States; ^3^ Laboratory of Cancer Biology and Genetics, National Cancer Institute, National Institutes of Health, Bethesda, MD, United States; ^4^ Oral Immunobiology Unit, National Institutes of Dental and Craniofacial Research, National Institutes of Health, Bethesda, MD, United States; ^5^ Division of Oral and Systemic Health Sciences, School of Dentistry, University of California, Los Angeles, Los Angeles, CA, United States

**Keywords:** mEAK-7, V-ATPase, lysosomal membrane protein, mTOR signaling, cancer

## Abstract

mEAK-7 (mammalian EAK-7 or MTOR-associated protein, eak-7 homolog), is an evolutionarily conserved lysosomal membrane protein that is highly expressed in several cancer cells. Multiple recent studies have identified mEAK-7 as a positive activator of mTOR (mammalian/mechanistic target of rapamycin) signaling via an alternative mTOR complex, implying that mEAK-7 plays an important role in the promotion of cancer proliferation and migration. In addition, structural analyses investigating interactions between mEAK-7 and V-ATPase, a protein complex responsible for regulating pH homeostasis in cellular compartments, have suggested that mEAK-7 may contribute to V-ATPase-mediated mTORC1 activation. The C-terminal α-helix of mEAK-7 binds to the D and B subunits of the V-ATPase, creating a pincer-like grip around its B subunit. This binding undergoes partial disruption during ATP hydrolysis, potentially enabling other proteins such as mTOR to bind to the α-helix of mEAK-7. mEAK-7 also promotes chemoresistance and radiation resistance by sustaining DNA damage-mediated mTOR signaling through interactions with DNA-PKcs (DNA-dependent protein kinase catalytic subunit). Taken together, these findings indicate that mEAK-7 may be a promising therapeutic target against tumors. However, the precise molecular mechanisms and signal transduction pathways of mEAK-7 in cancer remain largely unknown, motivating the need for further investigation. Here, we summarize the current known roles of mEAK-7 in normal physiology and cancer development by reviewing the latest studies and discuss potential future developments of mEAK-7 in targeted cancer therapy.

## Introduction

mEAK-7 (mammalian EAK-7 or MTOR-associated protein, eak-7 homolog), also known through genomics and proteomics studies as KIAA1609 (1, 2), LOC57707 (3), and TLDC1 (TBC/LysM-associated domain-containing 1) (4), is an evolutionarily conserved lysosomal membrane protein that promotes cell proliferation and migration (5). The mEAK-7 protein, first cloned and sequenced from a human fetal brain cDNA library, contains 473 amino acids and is ubiquitously expressed in all human adult and fetal tissues, with highest levels in the brain, kidney, spleen, and ovaries (1). The human ortholog *MEAK7* gene (NCBI Gene ID: 57707) is located at chromosome 16q24.1, and an average of 89% of amino acids are conserved in the mEAK-7 protein across eukaryotes (5). In *Caenorhabditis elegans*, EAK-7 (enhancer-of-*akt*-1-7), the mEAK-7 homologue in nematodes, nonautonomously controls both dauer arrest and longevity by negatively regulating the nuclear activity of the DAF-16/FoxO transcription factor in parallel to the insulin receptor signaling serine/threonine kinase AKT-1 (6). It has been shown that the mEAK-7 protein is expressed in the hippocampi of mouse brains, and while overexpression of mEAK-7 in N2a (Neuro 2a) cells does not reduce the level of oxidative stress *in vitro*, expression in mice reduces N2a cell death under oxidative stress conditions through an unknown mechanism (2).

Human mEAK-7 contains two domains: the TLDc (Tre2/Bub2/Cdc16 (TBC), lysine motif (LysM), domain catalytic) domain near the C-terminus and the N-myristoylation motif at the N-terminus (5). The TLDc domain is a highly conserved protein motif which plays an essential role in normal human brain development and is found in a family of five proteins which share protective functions against oxidative stress: NCOA7, TBC1D24, mEAK-7 (TLDC1), OXR1, and C20ORF118 (TLDC2) (2, 7, 8). Computational analysis predicts that mEAK-7 folds into α/β + β sheets to facilitate its enzymatic activity (5). In zebrafish, the crystal structure of the TLDc domain of oxidation resistance protein 2 forms two antiparallel β-sheets sandwiched between two helices and two one-turn helices (9). In humans, the 3-dimensional structure of the TLDc domain is globular and consists of two α-helices at the N-terminus and ten β-strands forming two antiparallel β-sheets (10). The structure of the TLDc domain does not resemble any other enzymes engaged in the protection against reactive oxygen species, and the intricate molecular mechanism underlying its antioxidant attributes remains unknown (11). The N-myristoylation motif anchors proteins to lipid bilayers and endomembrane compartments by irreversibly binding to myristate (12, 13).

Growing evidence indicates that mEAK-7 mediates the activation of the mTOR (mammalian/mechanistic target of rapamycin) pathway through an alternative mTOR complex (5, 14). mTOR regulates many fundamental cell processes including autophagy, apoptosis, cellular growth, proliferation, survival, metabolism, angiogenesis, transcription, and translation. Dysregulation and alterations in mTOR pathways lead to cancer development and advancement (15, 16). The mTOR complex involving mEAK-7 regulates S6K2 and 4E-BP1 in response to insulin, amino acids, and growth factors (5) and has also been shown to interact with V-ATPases (vacular-type adenosine triphosphatases, also known as the H^+^-ATPase) (8, 17, 18). Recent studies have found that human *MEAK7* mRNA is overexpressed in cancers such as hepatocellular carcinoma (19), lymph node-positive breast cancers (20), and non-small-cell lung cancer (14). Here, we present a concise overview of the latest significant discoveries concerning the physiological roles of mEAK-7 and current understanding in the context of carcinogenesis and the advancement of cancer.

## mEAK-7 and V-ATPase interaction

Recent findings demonstrate that all five members of the TLDc-containing proteins interact with V-ATPases (11). V-ATPases are conserved protein complexes that regulate pH homeostasis of intracellular compartments and organelles by catalyzing ATP hydrolysis to actively transport protons across biological membranes (21–24). V-ATPase-regulated pH acidification is essential for the normal physiological function of cells. Canonical roles attributed to V-ATPase include endocytic trafficking, protein processing and degradation, plasma membrane functions, and synaptic vesicle loading and coupled transport. Non-canonical functions of V-ATPase include membrane fusion, pH sensing, activation of mTORC1, and scaffolding protein-protein interactions (21). Dysregulated expression, misplacement, and genetic alterations affecting V-ATPases are intricately linked to cancer development and progression, making V-ATPases a central therapeutic target in translational medicine (22). Mammalian V-ATPases are composed of a cytosolic V_1_ domain (consisting of three copies each of the A, B, G, and E subunits and single copies of C, H, D, and F subunits), responsible for ATP hydrolysis, and a transmembrane V_0_ subcomplex (containing a single isoform of subunit a, 9 copies of c, and one copy each of d, e, and c’’) embedded in the lipid bilayer that participates in proton translocation (23). The c’ subunit within the V_0_ domain is only observed in fungi. The A_3_B_3_ subcomplex allows rotation of the rotor subcomplex, composed of subunits D, F, d, and the membrane-embedded c-ring (25). Rotation of the c-ring, comprised of nine c subunits and a single c″ subunit, against subunit “a” drives proton translocation through the membrane, and the reversible dissociation of the V_1_ and V_0_ regions stops the proton pumping (8, 26). The stationary component, relative to the rotor, has three peripheral stalks each composed of E and G heterodimers, enabling its ‘stator’ function primarily through the binding of C and H subunits of the V_1_ domain to subunits a of the V_0_ complex in a cohesive manner (26). In mammalian cells, there are several different isoforms of V-ATPase subunits (27); isoforms of the “a” subunit of the V_0_ domain contain location-specific information which is essential for directing V-ATPases to their target cellular compartments and creating specific pH values within them (22, 27).

**Figure 1:**
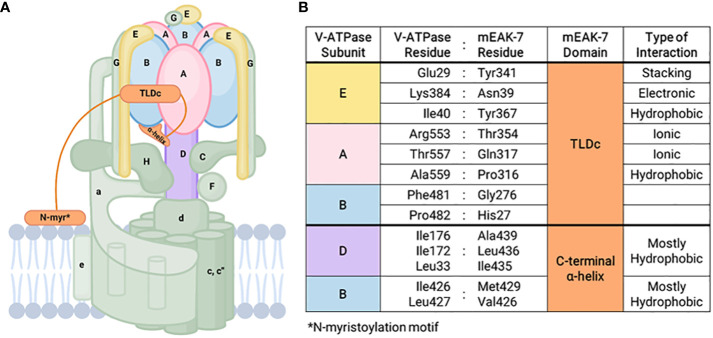
mEAK-7 interaction with V-ATPase. **(A)** Simplified structural diagram of V-ATPase and its interactions with mEAK-7. The C-terminal α-helix of mEAK-7 was shown to interact with the B and D subunits of V-ATPase, while the TLDc domain of mEAK-7 interacts with the E, A, and B subunits of V-ATPase. The N-myristoylation motif (denoted as “N-myr”) anchors proteins to lipid bilayers and endomembrane compartments by irreversibly attaching to myristate. **(B)** Specific amino acid interactions between residues in V-ATPase subunits and mEAK-7 domains presented by Tan et al. (8). These indicate potential sites for future targeted drug therapies.

Immunoprecipitation targeting the B subunit of V-ATPase mixed with the lysates from HEK293T cells overexpressing mEAK-7 revealed an interaction between mEAK-7 and the B subunit of V-ATPase (11). In addition, mEAK-7 associates with V-ATPases through its TLDc domain and C-terminal α-helix in rotational State 2 (**Figure 1**) (8). The C-terminal α-helix binds to the D and B subunits of the V-ATPase and crosslinks the stationary and rotor region of the enzyme through hydrophobic interactions, resulting in the formation of a pincer-like grip around the B subunit of V-ATPase (8, 28). Structural investigations also unveiled interactions between human mEAK7 and human V-ATPase, demonstrating an association between the TLDc domain of mEAK-7 with subunit A, B, and E of V-ATPase and the C-terminal domain of mEAK-7 with subunit D of V-ATPase (17). Another structural analysis complimented this finding by demonstrating that mEAK-7 interacts with subunits A, B, D, and E of V-ATPases in state 2 (18). Collectively, these findings suggest that mEAK-7 is a V-ATPase inhibitor, presumably blocking V_1_-V_0_ torque transmission. However, functional studies revealed that mEAK-7 does not affect the enzyme activity of V-ATPases and lysosomal or phagosomal pH *in vitro* (17, 28).

Bafilomycin, a V-ATPase blocker, inhibits lysosomal acidification, thereby preventing the activity of lysosomal proteases and autophagy by interfering with autophagosome-lysosome fusion (29). The overexpression of mEAK-7 also does not affect bafilomycin sensitivity, indicating that the V_1_ and V_0_ domains of V-ATPase may remain coupled even when mEAK-7 crosslinks the rotor and stator of V-ATPase. It is also possible that the direct crosslink between mEAK-7 and V-ATPase is interrupted during rotary catalysis (17, 18, 28). CryoEM studies of mEAK-7 and V-ATPase structural interactions showed a clear 3-dimensional structure in rotational State 2 before consumption of ATP, with no area that lacked density (8). However, when ATP was added, a population of particles associated with mEAK-7 in rotational State 2 and the density for the C-terminal α-helix disappeared, indicating that ATP hydrolysis might disrupt mEAK-7 and V-ATPase interactions (8).

## mEAK-7, V-ATPase, and the mTOR pathway

mTOR is a conserved serine/threonine kinase in the PI3K (phosphoinositide 3-kinases)-related kinase (PIKK) family and responds to nutrients and growth signals (16). mTOR forms two structurally and functionally distinct complexes known as mTORC1 (mTOR Complex 1) and mTORC2 (15, 16, 30). mTORC1 consists of mTOR, Raptor (regulatory-associated protein of mammalian target of rapamycin), mLST8 (mammalian lethal with Sec13 protein 8, also known as GβL), PRAS40 (proline-rich Akt substrate of 40 kDa), and DEPTOR (DEP domain containing mTOR-interacting protein) (30). mTORC2 consists of mTOR, Rictor (rapamycin-insensitive companion of mTOR), mLST8, DEPTOR, hSin1, and PRR5 (proline-rich protein 5), and predominantly regulates cytoskeletal structure, cellular metabolism, cell survival, and cell response to insulin (30–32). Recently, several studies demonstrated that mTOR activation requires localization to the lysosome surface (33, 34). When amino acids are available, cytosolic mTOR is recruited to the lysosome surface via Rag proteins (34). Upon growth factor stimulation, PI3K triggers the production of PIP_3_ in the plasma membrane, initiating a cascade to phosphorylate kinase Akt and its activator PDK1 (phosphoinositide-dependent protein kinase 1). This leads to the inhibition of the TSC (Tuberous sclerosis complex) and GAP (GTPase-activating protein) activity, culminating in an enhanced level of GTP-bound Rheb at the lysosome surface, where it directly binds and activates the mTORC1 complex (33–35). The activated mTORC1 has been known to phosphorylate both S6K1 (ribosomal protein S6 kinase) and 4E-BP (the eukaryotic initiation factor 4E binding protein), to promote cellular growth through protein anabolism (16, 36), nucleotide biosynthesis (37), lipogenesis (38), glycolysis (15, 16), and mitochondrial biogenesis (39).

Macrolide V-ATPase inhibitors effectively halt mTORC1 activation, indicating that V-ATPase affects activation of mTOR signaling (40). A small-molecule *in vivo* activator of autophagy, EN6, has been shown to covalently bind to Cysteine 277 in the ATP6V1A subunit of the lysosomal V-ATPase, decoupling it from RAGs and leading to the inhibition of mTORC1 signaling, enhancement of lysosomal acidification, and activation of autophagy (41). Conversely, mTORC1 has been shown to suppress V-ATPase activity by repressing V_1_-V_0_ assembly, enabling mTORC1 to respond promptly to changes in cellular nutrient environments by preventing lysosomal degradation of extracellular proteins (42). It has also been demonstrated that the late endosomal/lysosomal V-ATPase-Ragulator-RAG (Ras-related GTP binding) protein complex is required for mTORC1 activation (43). Upon high nutrient and energy levels, V-ATPase stimulates the GEF (guanine-nucleotide exchange factor) activity of Ragulator, consisting of LAMTOR1 (late endosomal/lysosomal adaptor, MAPK and mTOR activator 1) through LAMTOR5. This promotes the conversion of the GDP-bound form to the GTP-bound form of RAGA/RAGB, increasing the affinity of RAGs for mTORC1 (40, 44, 45). The RAG-bound mTORC1 is then activated after interacting with GTP-bound Rheb on the lysosomal surface (35). These findings highlight the essential role of V-ATPase activity in mTOR signaling and activation.

Previously, we demonstrated that mEAK-7 interacts with mTOR at the lysosome in response to nutrient stimulation and promotes cell proliferation and migration (5). In nematodes, EAK-7 is located in the plasma membrane, and there is some evidence that mEAK-7 can be detected at the plasma membrane in mammalian cells as well (5, 6). Research has shown that mEAK-7 predominantly binds to the lysosomal membrane. This is supported by the strong colocalization between mEAK-7 and LAMP1 (lysosomal-associated membrane protein-1) as well as LAMP2. In contrast, there is minimal to no colocalization observed in the endosome, mitochondria, endoplasmic reticulum, and golgi complex, indicating that mEAK-7’s association is primarily with the lysosomal membrane (5). The knockdown of mEAK-7 in several human cancer cell lines with high endogenous mEAK-7 expression, such as H1975 (non-small cell lung cancer, NSCLC), H1299 cells (NSCLC), and MDA-MB-231 (breast cancer), resulted in decreased lysosomal localization of mTOR, marked attenuation of S6K2 and 4E-BP1 phosphorylation, and diminished cell proliferation and migration. These effects were rescued upon overexpression of mEAK-7, which reactivated mTOR signaling (5). Furthermore, the TLDc domain and C-terminus of mEAK-7 are crucial for facilitating mTOR kinase activity, and although mEAK-7 interacts with mTOR and mLST8, it does not engage with Raptor or Rictor, key constituents of the mTORC1 and mTORC2 complexes (5). Taken together, these findings suggest that mEAK-7 might play a key role in recruiting mTOR to the lysosome and activating mTOR kinase function via an alternative mTOR complex in mammalian cells.

**Figure 2:**
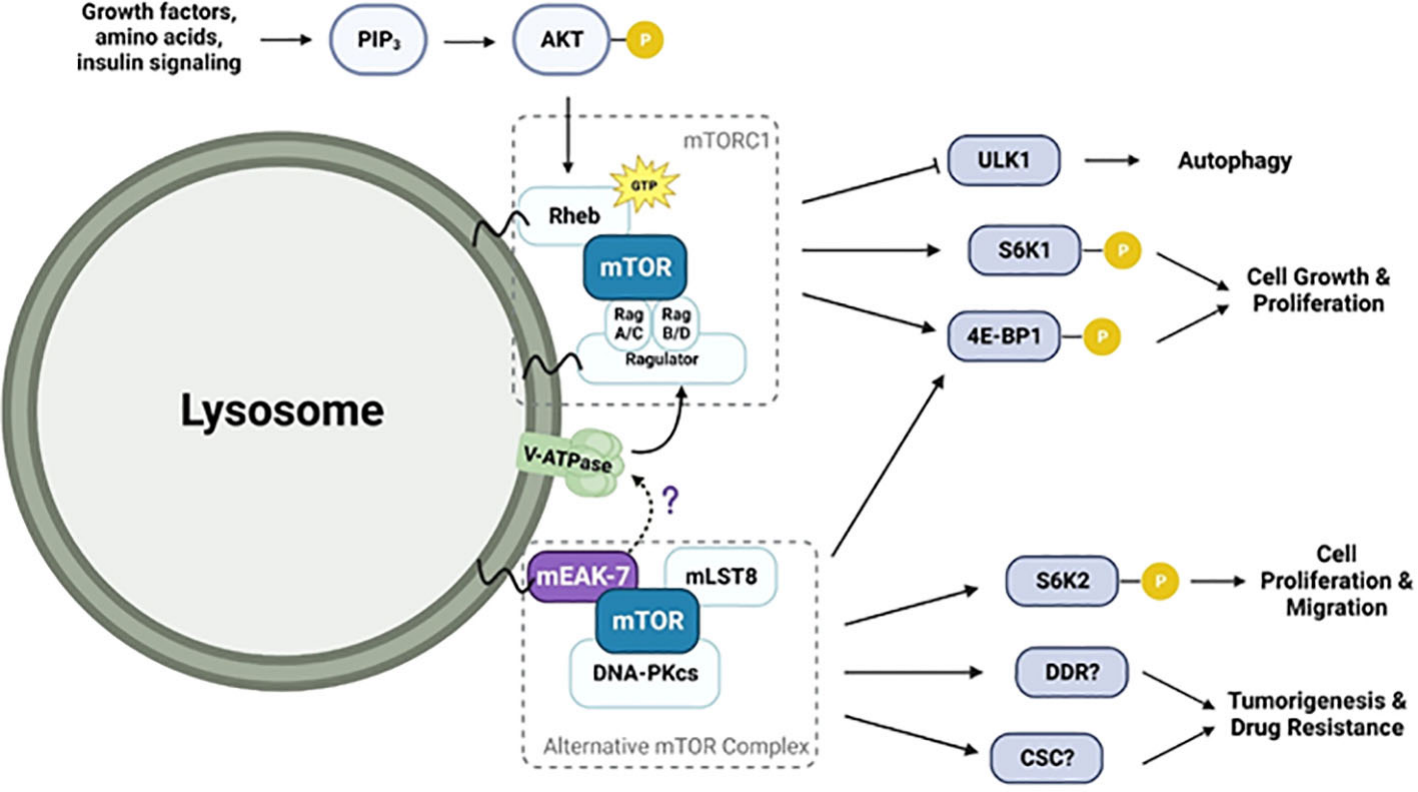
Diagram illustrating the potential mEAK-7-mediated mTOR activation pathway and their interactions with V-ATPase. V-ATPase stimulates GEF activity of Ragulator, promoting conversion of GDP to GTP-bound RAGs and increasing their affinity for mTORC1. RAG-bound mTORC1 is then activated after interacting with GTP-bound Rheb. mEAK-7 interacts with mTOR to form an alternative mTOR complex involving mLST8 and DNA-PKcs. This complex is known to play a role in phosphorylating S6K2 and 4E-BP1, leading to cell growth, proliferation, and migration. Although structural studies have suggested that mEAK-7 interacts with V-ATPase, the precise mechanism is still unknown.

As discussed above, numerous studies have investigated interactions among mEAK-7, V-ATPase, and mTORC1. Although the exact nature of these interactions remains unclear, the discoveries made so far are as follows: 1) The mEAK-7 and mTORC1 have physical and functional interactions; 2) The interaction between mEAK-7 and the V-ATPase is predicted to be inhibitory; however, this interaction could be disrupted by ATP hydrolysis, potentially liberating part of mEAK-7 to serve as a binding site for other molecules. There is speculation that mTORC1 might bind to the released segment of mEAK-7, although empirical evidence to support this interaction remains elusive; and 3) The interaction between V-ATPase and mTORC1 exhibits significant complexity. V-ATPase interactions with Rag proteins are required for mTORC1 activity (40), but inhibition of mTORC1 activates V-ATPase by both increasing its assembly (42) and enhancing synthesis via the TFEB pathway (46). Furthermore, despite the interactions between each pair, currently, there is limited data supporting simultaneous physical or functional interactions amongst all three of the molecules (mEAK-7, mTORC1, and V-ATPase). Also, the precise role mEAK-7 may play in such interactions remains unclear. Recently, structural investigations have unveiled the potential involvement of mEAK-7 in V-ATPase-mediated mTOR signaling (8, 17, 18). While it was initially suggested that the C-terminal α-helix of mEAK-7 could engage with mTOR (5), this interaction through the α-helix seems unfeasible when mEAK-7 is bound to V-ATPase. However, a structural and functional investigation showed that the interaction between mEAK-7 and V-ATPase, hinged on the C-terminal α-helix, undergoes partial disruption during ATP hydrolysis (8, 28). This potentially enables binding of other proteins (47). Alternatively, this mechanism might serve to regulate binding of mEAK-7 to both V-ATPase and other proteins (8). Furthermore, one recent study suggests that mEAK-7 may interfere with V-ATPase-mTOR signaling (17). This validation involved the generation of a stable HCT116 cell line (human colorectal carcinoma) that constitutively expresses the α4 subunit of V-ATPase (HCT-a4), which localizes to the lysosome. Western blot analysis demonstrated that after treatment with bafilomycin A1, the phosphorylation of S6 protein at Ser^235/236^ was completely abolished. In addition, overexpression of mEAK7 in HC6-a4 cells inhibited the phosphorylation of S6 at Ser^235/236^ and 4E-BP1 at Ser^65^, indicating that mEAK7 may interfere with the V-ATPase-mTOR signaling (17). However, the authors of this study explicitly stated that this outcome might be due to using a ‘special class of cells’ with V-ATPase overexpression. **Figure 2** illustrates the potential pathways involving mTORC1, V-ATPase, and the alternative mTOR complex. Collectively, these findings reinforce the need for further studies aimed at unraveling the details of mEAK-7-mediated regulation within the context of V-ATPase-mediated mTOR signaling or mEAK-7-mediated mTOR signaling, while also delving into the prospective contributions of mEAK-7 in other pathways, potentially mediated by its interactions involving the α-helix.

## mEAK-7 and cancer

As previously explored, mEAK-7 plays a crucial role in activating the mTOR pathway that regulates cellular growth and migration, maintains physiological hemostasis, and orchestrates metabolism in response to external stimuli (16). The dysregulation of these processes contributes to the development and progression of pathophysiological conditions such as cancer and neurological disorders (5, 14, 15). Heightened mRNA expression levels of mEAK-7 have been observed in several cancer cell lines (5) and there is significant elevation of both mEAK-7 and mTOR signaling in tumor and metastatic lymph nodes of patients diagnosed with NSCLC (14). In two well-established NSCLC cell lines, H1975 and H1299, stem-like CD44^+^/CD90^+^ cells, which represented 1% of the total cell population, yielded elevated protein levels of mEAK-7, S6K2, n-cadherin (marker for the epithelial-mesenchymal transition state in cancer stem cells), and phosphorylated S6 and 4E-BP1, indicating activated mTOR signaling with higher invasive potential compared to CD44^-^/CD90^-^ cells (14). Furthermore, mEAK-7 is necessary for clonogenic potential and spheroid formation of CD44^+^/CD90^+^ NSCLS cells and promotion of cisplatin- and radiation resistance through DNA damage-mediated mTOR signaling, in part through interaction with DNA-PKcs (DNA-dependent protein kinase catalytic subunit) (14, 48). Knockdown of mEAK-7 impairs the DNA damage response and enhances proapoptotic Noxa levels and PARP (poly (ADP-ribose) polymerase) cleavage in cancer cells. A bioinformatic search using the Exiqon miRSearch V3.0 algorithm revealed that MicroRNA-1911-3p regulates mEAK-7 translation (49). The study revealed that MicroRNA-1911-3p targets mEAK-7 mRNA at 3’UTR and decreases mEAK-7 protein levels, leading to suppressed mTOR signaling, which was evidenced by significantly decreased mTOR-dependent S6 and 4E-BP1 phosphorylation in NSCLC cell lines. Furthermore, H1299 cells transfected with a MicroRNA-1911-3p mimic significantly decreased mTOR and LAMP2 colocalization, demonstrating that repressed mEAK-7 levels impair co-localization of mTOR to the lysosome and subsequently attenuates NSCLC cell proliferation and migration. Collectively, these studies provide a novel perspective towards developing a cancer treatment targeting mEAK-7-associated mTOR signaling in NSCLC and other malignancies exhibiting elevated mEAK-7 expression.

## Conclusion

The lysosome-bound mEAK-7 protein plays an important role in the intricate regulation of cellular proliferation and migration by activating mTOR via an alternative pathway and/or interacting with V-ATPase to modulate V-ATPase-mediated mTOR activation. Structural and functional analyses conducted in both physiological and pathophysiological conditions suggest mEAK-7 could serve as a promising therapeutic target against tumors, particularly non-small-cell lung cancer. However, despite recent findings, the precise molecular mechanisms and signal transduction pathways controlled by mEAK-7 in cancer remain largely unknown, with *in vivo* investigations yet to be systematically explored. The elucidation of the mEAK-7 protein structure and its interaction with V-ATPases presents a remarkable opportunity for the development of a potent mEAK-7 inhibitor with specific binding characteristics while minimizing adverse effects, paving the way for the generation of mEAK-7-targeted therapy in cancer. This exciting potential not only broadens the current understanding of cancer biology but also highlights the possibility of tailored interventions that could revolutionize treatment strategies.

## Author contributions

IC: Writing – original draft, Writing – review & editing. YL: Writing – original draft, Writing – review & editing. JP: Writing – review & editing. JN: Writing – review & editing. JK: Writing – review & editing. PK: Writing – review & editing.
